# Targeting the ACOD1-itaconate axis stabilizes atherosclerotic plaques

**DOI:** 10.1016/j.redox.2024.103054

**Published:** 2024-01-22

**Authors:** Karl J. Harber, Annette E. Neele, Cindy PAA. van Roomen, Marion JJ. Gijbels, Linda Beckers, Myrthe den Toom, Bauke V. Schomakers, Daan AF. Heister, Lisa Willemsen, Guillermo R. Griffith, Kyra E. de Goede, Xanthe AMH. van Dierendonck, Myrthe E. Reiche, Aurélie Poli, Frida L-H Mogensen, Alessandro Michelucci, Sanne GS. Verberk, Helga de Vries, Michel van Weeghel, Jan Van den Bossche, Menno PJ. de Winther

**Affiliations:** a Department of Medical Biochemistry, Amsterdam UMC, University of Amsterdam, 1105 AZ, Amsterdam, the Netherlands; b Amsterdam Cardiovascular Sciences (ACS), Atherosclerosis & Ischemic Syndromes, Amsterdam UMC, the Netherlands; c Amsterdam Institute for Infection and Immunity (AII), Inflammatory Diseases, Amsterdam UMC, the Netherlands; d Department of Molecular Cell Biology and Immunology, Amsterdam UMC, Vrije Universiteit Amsterdam, 1081 HV, Amsterdam, the Netherlands; e Department of Pathology, CARIM, Cardiovascular Research Institute Maastricht, GROW-School for Oncology and Developmental Biology, Maastricht UMC, University of Maastricht, 6229 HX, Maastricht, the Netherlands; f Department of Genetic Metabolic Diseases, Amsterdam UMC, University of Amsterdam, 1105 AZ, Amsterdam, the Netherlands; g Amsterdam Gastroenterology Endocrinology Metabolism (AGEM), Amsterdam UMC, the Netherlands; h Department of Medical Cell Biology, Uppsala University, 75236, Uppsala, Sweden; i Neuro-Immunology Group, Department of Cancer Research, Luxembourg Institute of Health, 6A Rue Nicolas-Ernest Barblé, L-1210, Luxembourg, Luxembourg; j Amsterdam Neuroscience, Amsterdam, the Netherlands; k Core Facility Metabolomics, Amsterdam UMC, University of Amsterdam, 1105 AZ Amsterdam, the Netherlands

**Keywords:** Itaconate, IRG1, Acod1, Immunometabolism, Atherosclerosis, Macrophage

## Abstract

Inflammatory macrophages are key drivers of atherosclerosis that can induce rupture-prone vulnerable plaques. Skewing the plaque macrophage population towards a more protective phenotype and reducing the occurrence of clinical events is thought to be a promising method of treating atherosclerotic patients. In the current study, we investigate the immunomodulatory properties of itaconate, an immunometabolite derived from the TCA cycle intermediate *cis*-aconitate and synthesised by the enzyme Aconitate Decarboxylase 1 (ACOD1, also known as IRG1), in the context of atherosclerosis. *Ldlr*^−/−^ atherogenic mice transplanted with *Acod1*^−/−^ bone marrow displayed a more stable plaque phenotype with smaller necrotic cores and showed increased recruitment of monocytes to the vessel intima. Macrophages from *Acod1*^*−/−*^ mice contained more lipids whilst also displaying reduced induction of apoptosis. Using multi-omics approaches, we identify a metabolic shift towards purine metabolism, in addition to an altered glycolytic flux towards production of glycerol for triglyceride synthesis. Overall, our data highlight the potential of therapeutically blocking ACOD1 with the aim of stabilizing atherosclerotic plaques.

## Graphical abstract

**Figure:**
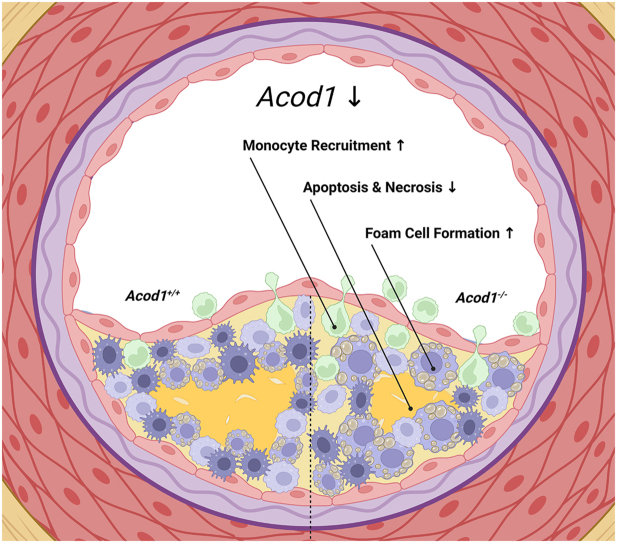

## 1 Introduction

Atherosclerosis is the leading cause of cardiovascular disease (CVD) and consequently a global health concern, where CVD is responsible for over 30 % of deaths worldwide. Use of cholesterol-lowering interventions such as statins and PCSK9 inhibitors, although effective in lowering low-density lipoprotein (LDL) cholesterol, comes with numerous side effects and does not address the residual inflammatory risk of CVD patients [[1], [2], [3], [4]]. In atherosclerosis patients, lipid deposition and subsequent oxidation in vessel sub-endothelium initiates recruitment of monocytes to the lesion area, developing into mature macrophages [5,6]. As such, macrophages attempt to clear modified lipids by scavenger receptor-mediated endocytosis and therefore become lipid-laden foam cells (FCs), a characteristic cell type of atherosclerosis. Dysregulated lipid handling of FCs ultimately leads to cell necrosis and subsequent plaque growth [7]. Development of the necrotic core drives further recruitment of monocytes and release of pro-inflammatory mediators, generating unstable plaques that may rupture, leading to thrombus formation and subsequent infarction [8]. Contrary to this, anti-inflammatory macrophages are critical in stabilizing plaques through necrotic core maintenance and fibrous cap thickening [9]. Promoting an increased ratio of anti-inflammatory to pro-inflammatory macrophage subsets thus may improve plaque stability whilst also tackling residual inflammation in atherosclerosis patients. As such, monoclonal antibodies targeting interleukin-1 beta (IL-1β) in the CANTOS trial resulted in lower cardiovascular events, however total mortality was unaffected due to severe leukopenia and subsequent sepsis [10]. Therefore, new combined methods of specifically reducing plaque inflammation and LDL content are needed for successful reduction of CVD related deaths [11].

In the last 10 years it has been unveiled that metabolites have a broader function outside of their energetic roles [12]. Upon inflammatory activation, macrophages undergo vast re-wiring of their TCA cycle, resulting in accumulation or depletion of certain metabolites. Such alterations in metabolite levels can elicit functional changes through receptor binding, post-translational modifications or act as co-factors for enzymes [[13], [14], [15]]. Itaconate is a macrophage-specific, inflammation-induced derivative of the TCA cycle intermediate *cis*-aconitate, produced by the enzyme *cis*-aconitate-decarboxylase (encoded by *ACOD1*, also known as IRG1), formed from the metabolite *cis*-aconitate [16]. Upon LPS stimulation, *ACOD1* and itaconate are one of the most upregulated genes and metabolites in both human and mouse macrophages. Functional testing of itaconate and its derivatives on macrophages identified these molecules as potent regulators of inflammatory signaling and redox biology [[16], [17], [18], [19], [20]]. In parallel, *Acod1*^−/−^ bone marrow-derived macrophages (BMDMs), depleted of endogenous itaconate, display significantly higher inflammatory responses than their *Acod1*^+/+^ BMDMs counterparts [17]. Moreover, Acod1 has been shown to modulate the generation of reactive oxygen species (ROS), a key player in the development of atherosclerotic plaques [[21], [22], [23], [24]]. As such, understanding the effects of itaconate on the handling of excessive lipids, oxidative stress and inflammatory responses during atherogenesis could help harness control over immune cell-driven CVD and form the basis for novel therapeutic options [25,26].

In this paper, we explore *Acod1* as a modulator of atherosclerosis progression. We show that *Acod1-*deficiency increases plaque stability and reduces necrosis in the *Ldlr*^*−/−*^ atherosclerosis mouse model, as well as increasing macrophage viability and lipid content.

## 2 Results

### 2.1 Transplantation of Acod1^−/−^ bone marrow cells increases circulating Ly6C^low^ monocytes in an atherosclerosis mouse model

**Fig. 1:**
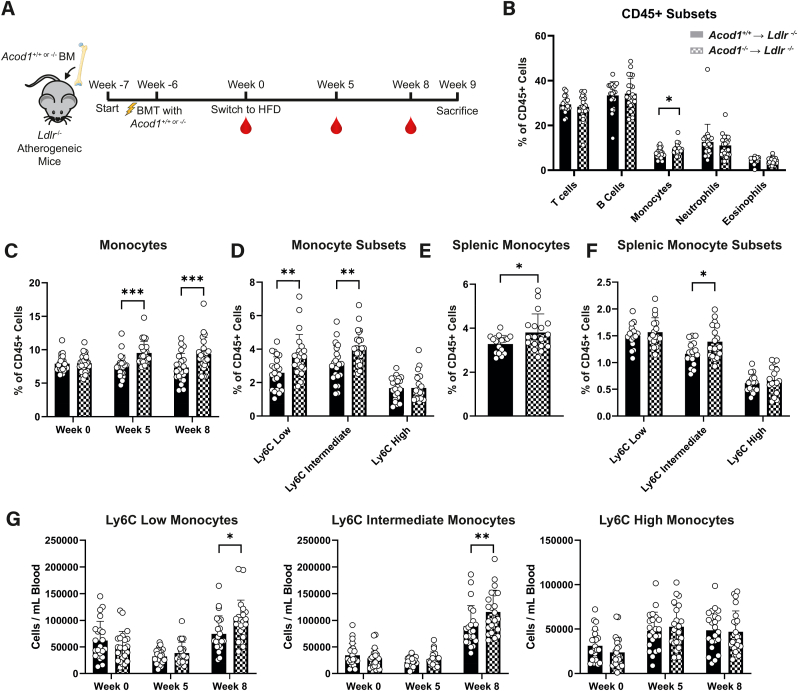
***Ldlr***^**−/−**^**mice transplanted with *Acod1***^**−/−**^**bone marrow contain more circulating and splenic Ly6c**^**Low**^**patrolling monocytes. A)** Irradiated atherosclerosis-susceptible *Ldlr*^−/−^ mice were transplanted with either *Acod1*^+/+^ or *Acod1*^−/−^ bone marrow (BM) and were given 6 weeks for efficient engraftment prior to initiating a high-fat diet (HFD). Blood samples are taken on weeks 0, 5 and 8 of HFD and mice were sacrificed on week 9. **B)** leukocyte subsets in *Acod1*^+/+^ → *Ldlr*^*−/−*^ (filled) or *Acod1*^−/−^ → *Ldlr*^*−/−*^ (checkered) mice at week 8 expressed as a percentage of circulating CD45^+^ cells (*n* = 22/23 for *Acod1*^+/+^ → *Ldlr*^*−/−*^/*Acod1*^−/−^ → *Ldlr*^*−/−*^, unpaired *t*-test). **C)** Blood monocytes in *Acod1*^+/+^ → *Ldlr*^*−/−*^ (filled) or *Acod1*^−/−^ → *Ldlr*^*−/−*^ (checkered) mice at week 0, 5 and 8 expressed as a percentage of circulating CD45^+^ cells (*n* = 22/23 for *Acod1*^+/+^ → *Ldlr*^*−/−*^/*Acod1*^−/−^ → *Ldlr*^*−/−*^, two-way ANOVA). **D)** Blood Ly6C monocyte subsets in *Acod1*^+/+^ → *Ldlr*^*−/−*^ (filled) or *Acod1*^−/−^ → *Ldlr*^*−/−*^ (checkered) mice at week 8 expressed as a percentage of circulating CD45^+^ cells (*n* = 22/23, two-way ANOVA). **E)** Splenic monocytes in *Acod1*^+/+^ → *Ldlr*^*−/−*^ (filled) or *Acod1*^−/−^ → *Ldlr*^*−/−*^ (checkered) BM expressed as a percentage of splenic CD45^+^ cells (*n* = 17/18, unpaired *t*-test). **F)** Splenic Ly6C monocyte subsets in *Acod1*^+/+^ → *Ldlr*^*−/−*^ (filled) or *Acod1*^−/−^ → *Ldlr*^*−/−*^ (checkered) mice expressed as a percentage of splenic CD45^+^ cells (*n* = 17/18, two-way ANOVA). **G)** Ly6C low (left), intermediate (middle) and high (right) counts per mL of blood in *Acod1*^+/+^ → *Ldlr*^*−/−*^ (filled) or *Acod1*^−/−^ → *Ldlr*^*−/−*^ (checkered) mice at weeks 0, 5 and 8 of HFD (*n* = 22/23, two-way ANOVA). Gating strategies for flow cytometry is provided in Supplemental Fig. 3. Data points display individual mice and error bars show SD.

**Supplemental Fig. 1:**
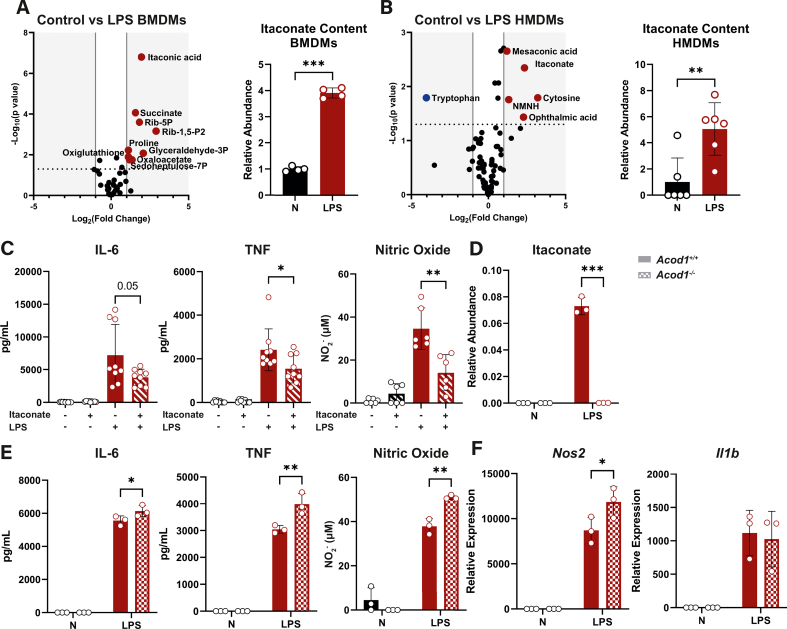
**Confirmation of previously published properties itaconate and itaconate deficiency have on BMDMs. A)** Metabolomics data shown in a volcano plot with increased (red) or decreased (blue) metabolites and quantified itaconate content in a bar graph, of BMDMs (*n* = 4, unpaired *t*-test) or **B)** human macrophages s (*n* = 6, paired *t*-test) treated with LPS for 24 h compared to unstimulated cells. Dotted line represents significance cut-off (p < 0.05). **C)** Secretion of IL6 (left), TNF (middle) and nitric oxide (right) after treatment with (stripes) or without (filled) itaconate (5 mM) for 3 h before stimulation with (red) or without (black) LPS for 24 h (*n* = 9 for ELISA, *n* = 6 for NO assay, unpaired *t*-test). **D)** Itaconate content from metabolomics of *Acod1*^+/+^ (filled) and *Acod1*^−/−^ (checkered) BMDMs treated with LPS for 24 h or control (*n* = 3, two-way ANOVA). **E)** Secretion of cytokines in control and LPS-treated *Acod1*^+/+^ and *Acod1*^−/−^ macrophages after 24 h measured by either ELISA or NO assay and **F)** inflammatory gene expression after 3 h of LPS (*n* = 3, two-way ANOVA). Data points display individual mice and error bars show SD.

**Supplemental Fig. 2:**
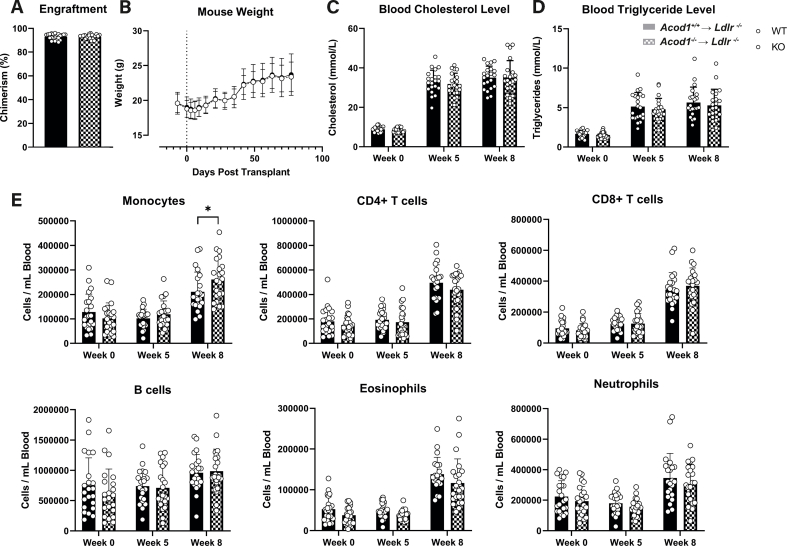
***In vivo* mouse and blood measurements. A)** Percentage chimerism of donor bone marrow measured by qPCR analysis of *Ldlr* expression in blood samples of *Acod1*^+/+^ → *Ldlr*^*−/−*^ (filled) and *Acod1*^−/−^ → *Ldlr*^*−/−*^ (checkered) mice (*n* = 22/23 for *Acod1*^+/+^ → *Ldlr*^*−/−*^ /*Acod1*^−/−^ → *Ldlr*^*−/−*^). **B)** Mouse weight over the entire *in vivo* experiment starting from 1 week before BMT in *Acod1*^+/+^ → *Ldlr*^*−/−*^ (black dots) and *Acod1*^−/−^ → *Ldlr*^*−/−*^ (white dots) mice (*n* = 22/23; dotted line is day of BMT). **C)** Cholesterol and **D)** triglyceride levels in blood plasma of mice at weeks 0, 5 and 8 of HFD (*n* = 22/23). **E)***In vivo* blood cell subset composition per mL of blood at weeks 0, 5 and 8 of HFD (*n* = 22/23). Data points display individual mice and error bars show SD. *BMT* = Bone Marrow transplantation; *HFD* = High Fat Diet.

**Supplemental Fig. 3:**
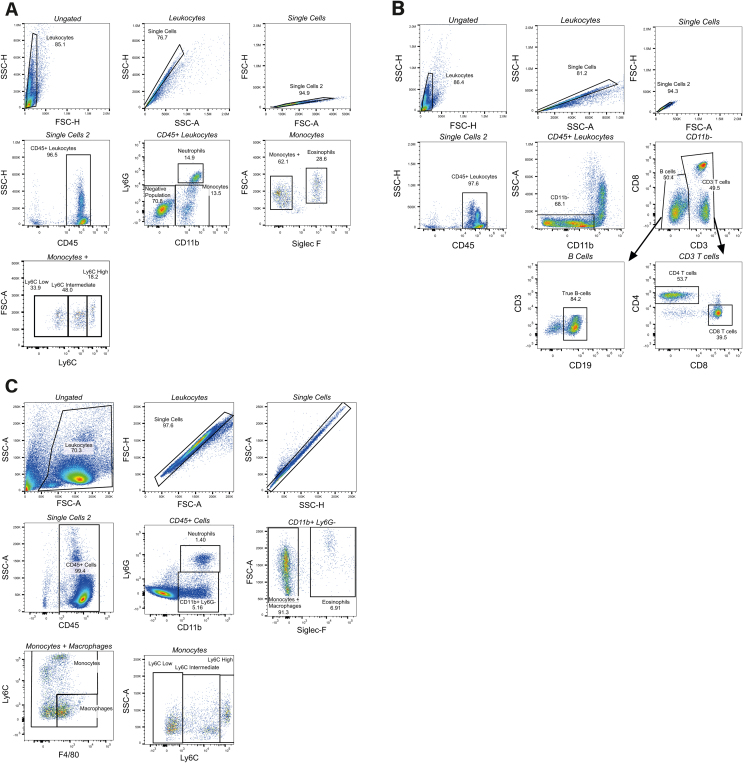
**Gating strategies for *in vivo* blood and spleen flow cytometry analysis. A)** Example gating strategies for myeloid and **B)** lymphoid cell subsets during blood collections at week 0, 5 and 8 of HFD. **C)** Example flow cytometry gating for dissociated spleen samples on day of sacrifice. All plots are read from left to right and top to bottom unless highlighted by a black arrow. Italic plot titles highlight which gate is being displayed from the previous plot.

Since targeting the Acod1-itaconate axis could be a promising modulator of atherosclerosis progression, we first reassessed itaconate's immunomodulatory properties. In this study, we used LPS to activate macrophages via TLR4 to elicit itaconate production for *in vitro* functional studies. We consider this an appropriate model to complement our *in vivo* atherosclerosis studies since (i) TLR4 and its' signalling pathways play a major role in atherosclerosis progression and (ii) atherosclerosis-relevant stimuli like oxLDL do not induce itaconate *in vitro* (data not shown) [[27], [28], [29]]. We confirmed accumulation of itaconate upon inflammatory activation in both mouse and human macrophages by metabolomics Supp. Fig. 1A and B) as previously described [16,17,19]. Additionally, we confirmed the anti-inflammatory effects of the natural unmodified form of itaconate by measuring cytokine secretion (Supp. Fig. 1C). Next, we verified itaconate deficiency in *Acod1*^−/−^ BMDMs (Supp. Fig. 1D), which increased pro-inflammatory responses compared to *Acod1*^+/+^ BMDMs at the protein and genetic level (Supp. Fig. 1E and F). After confirmation that unmodified itaconate and *Acod1* deficiency modulate macrophage inflammation, we investigated the role of Acod1-derived itaconate in a mouse model for atherosclerosis. Here, we transplanted bone marrow from either *Acod1*^+/+^ or *Acod1*^−/−^ mice into lethally irradiated atherosclerosis-susceptible *Ldlr*^−/−^ mice (*Acod1*^+/+^ → *Ldlr*^*−/−*^ and *Acod1*^−/−^ → *Ldlr*^*−/−*^, respectively) (Fig. 1A). Successful engraftment was confirmed prior to switching to a high fat diet with 0.125 % cholesterol (HFD) (Supp. Fig. 2A). All mice showed a similar increase in plasma cholesterol, triglyceride levels, and body weight (Supp. Figs. 2B–D). In addition, both groups displayed similar blood leukocyte composition except monocyte percentage which was elevated in the blood of mice transplanted with *Acod1*^−/−^ BM when compared to mice transplanted with *Acod1*^+/+^ BM (Fig. 1B and C & Supp. Fig. 2E, 3A & 3B). Ly6C^low^ and Ly6C^int^ subsets were responsible for the increase (Fig. 1D). The raised monocyte content was further supported by increased percentage of splenic monocytes (Fig. 1E & 1F & Supp. Fig. 3C) and monocytosis of Ly6C^low^ and Ly6C^int^ monocytes in *Acod1*^−/−^ → *Ldlr*^*−/−*^ mice 8 weeks after HFD. This suggests that the observed differences in monocyte abundance and composition between *Acod1*^+/+^ → *Ldlr*^*−/−*^ and *Acod1*^−/−^ → *Ldlr*^*−/−*^ mice were not due to differences in BM composition prior to transplantation, but rather occurred upon diet switching and atherosclerosis progression (Fig. 1G). Consequently, transplantation of *Acod1*-deficient bone marrow leads to blood monocytosis skewed in the direction of patrolling Ly6C^low^ monocytes.

### 2.2 Acod1^−/−^ monocytes show enhanced migration to atherosclerotic plaques

**Fig. 2:**
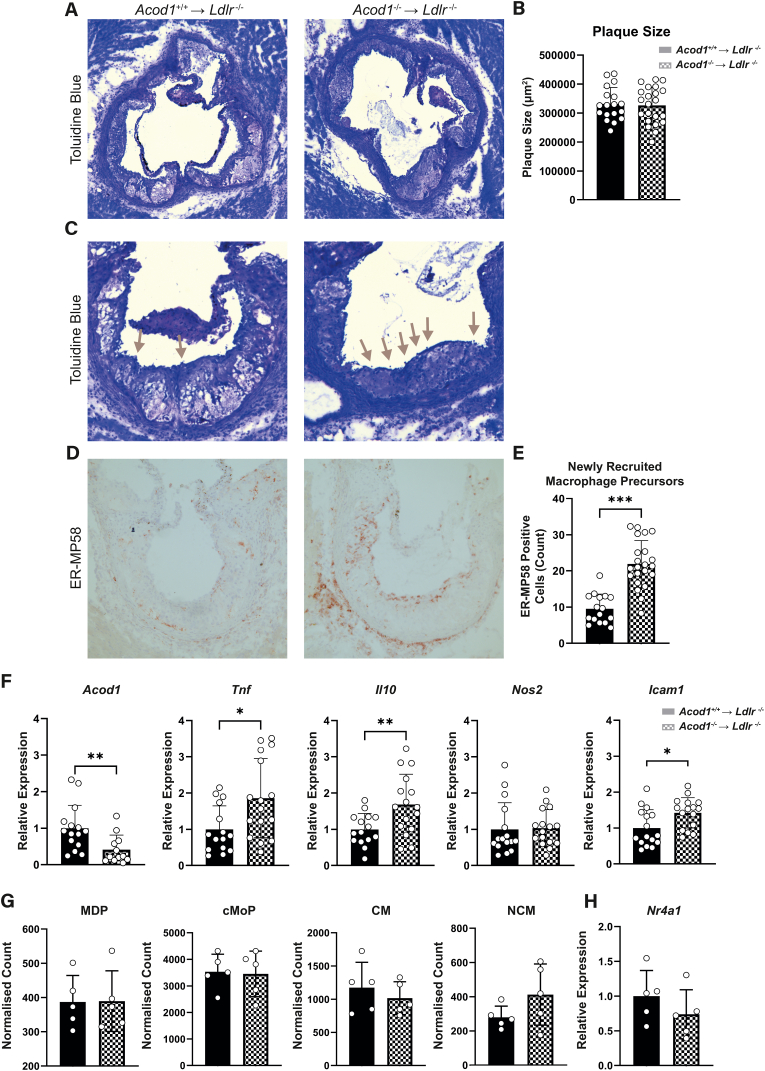
***Acod1***^**−/−**^**transplanted *Ldlr***^**−/−**^**mice have increased monocyte recruitment to the plaque. A)** Aortic root segments were taken from *Acod1*^+/+^ → *Ldlr*^*−/−*^ (left) and *Acod1*^−/−^ → *Ldlr*^*−/−*^ (right) mice and stained with toluidine blue to **B)** quantify total plaque size of all 3 valve cusps (*n* = 18/22 for *Acod1*^+/+^ → *Ldlr*^*−/−*^/*Acod1*^−/−^ → *Ldlr*^*−/−*^, unpaired *t*-test). **C)** One of the three valve cusps from the toluidine blue staining displaying luminal adhesion of monocytes (grey arrows). **D)** Immunohistochemical staining of monocyte antigen ER-MP58 (brown) which was then **E)** quantified by counting the number of positive cells on the luminal endothelium and within the plaque (*n* = 16/22, unpaired *t*-test). **F)** Gene expression of inflammatory and migration markers from mouse aortic arches (*n* = 15/16, unpaired *t*-test). **G)** Flow cytometry quantification of bone marrow myeloid precursors normalized to CD45^+^ count (*n* = 5, unpaired *t*-test). **H)** qPCR-derived gene expression of monocyte developmental marker *Nr4a1* in isolated peritoneal foam cells (FCs) (*n* = 5, unpaired *t*-test). Gating strategies for flow cytometry is provided in Supplemental Fig. 4. Data points display individual mice and error bars show SD. *MDP* = Monocyte and Dendritic Progenitor; *CMP* = Common Monocyte Progenitor; *CM* = Classical Monocyte; *NCM* = Non-Classical Monocyte. (For interpretation of the references to colour in this figure legend, the reader is referred to the Web version of this article.)

**Supplemental Fig. 4:**
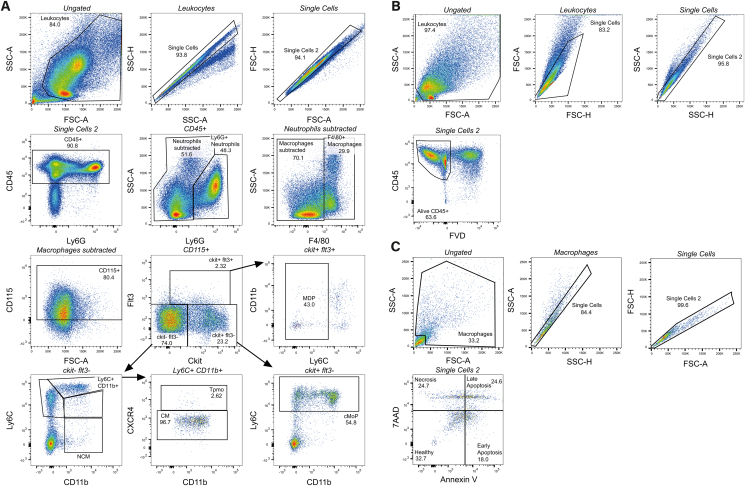
**Peritoneal foam cells from both *Acod1***^**+/+**^**and *Acod1***^**−/−**^**transplanted mice have the same cytokine secretion potential. A)** Cytokine secretion from *Acod1*^+/+^ → *Ldlr*^*−/−*^ (filled) and *Acod1*^−/−^ → *Ldlr*^*−/−*^ (checkered) FCs stimulated with LPS for 24 h measured by either ELISA or **B)** NO assay (*n* = 5/4 for *Acod1*^+/+^/*Acod1*^−/−^, unpaired *t*-test). Data points display individual mice and error bars show SD.

To assess the effect of *Acod1* deficiency on plaque phenotype, hearts were removed upon sacrifice to perform histological staining of the aortic root. Measurement of the total plaque size revealed no difference between Acod1^+/+^ → Ldlr^−/−^ and Acod1^−/−^ → Ldlr^−/−^ groups (Fig. 2A & B). Examining the plaques of the aorta, we observed higher quantities of adherent cells attached to the endothelium in Acod1^−/−^ → Ldlr^−/−^ compared to Acod1^+/+^ → Ldlr^−/−^ mice, which was in line with the increase in circulating blood monocytes (Fig. 2C). We next performed an IHC staining of monocyte-specific early recruitment marker ER-MP58 and quantified the number of positive monocytes, which showed significantly more ER-MP58^+^ monocytes in the Acod1^−/−^ → Ldlr^−/−^ group (Fig. 2D & E), indicating enhanced monocyte recruitment [30]. In order to further understand why Acod1^−/−^ → Ldlr^−/−^ mice have increased circulating monocytes and recruitment, we measured the expression of inflammatory and transmigration genes within aortic arches (Fig. 2F). As expected, Acod1^+/+^ → Ldlr^−/−^ control arches showed higher Acod1 expression than Acod1^−/−^ → Ldlr^−/−^ arches, highlighting the presence of Acod1-positive bone marrow-derived cells in control atherosclerotic plaques and confirming effective transplantation of Acod1 deficient bone marrow. Reduced Acod1 expression in the Acod1^−/−^ → Ldlr^−/−^ arch*es* resulted in increased expression of the pro-inflammatory marker *Tnf* and the anti-inflammatory marker *Il10* as well as endothelial transmigration gene *Icam1*. Expression of *Nos2*, which encodes the enzyme inducible nitric oxide synthase, was similar between the two groups. Subsequently, we measured the content of monocyte and dendritic progenitors (MDPs) and common monocyte progenitors (CMPs) in the bone marrow of these mice. Bone marrow precursor populations, as well as bone marrow classical and non-classical monocytes, however, were unaffected between the groups (Fig. 2G & Supp. Fig. 4A). Since bone marrow precursor proliferation could not explain the observed monocytosis, we analyzed the expression of pro-Ly6C^low^ transcription factor *Nr4a1* (Nur77). However, expression of *Nr4a1* was also unaffected in *Acod1*^−/−^ → *Ldlr*^*−/−*^ macrophages (Fig. 2H). With this data, we show that blood monocytosis and increased monocyte recruitment to the plaque is not due to increased bone marrow proliferation, but could be pertained to the altered expression of inflammatory mediators such as *Tnf* and *Icam1*.

### 2.3 Acod1-deficient atherogenic mice show increased foam cell formation

**Fig. 3:**
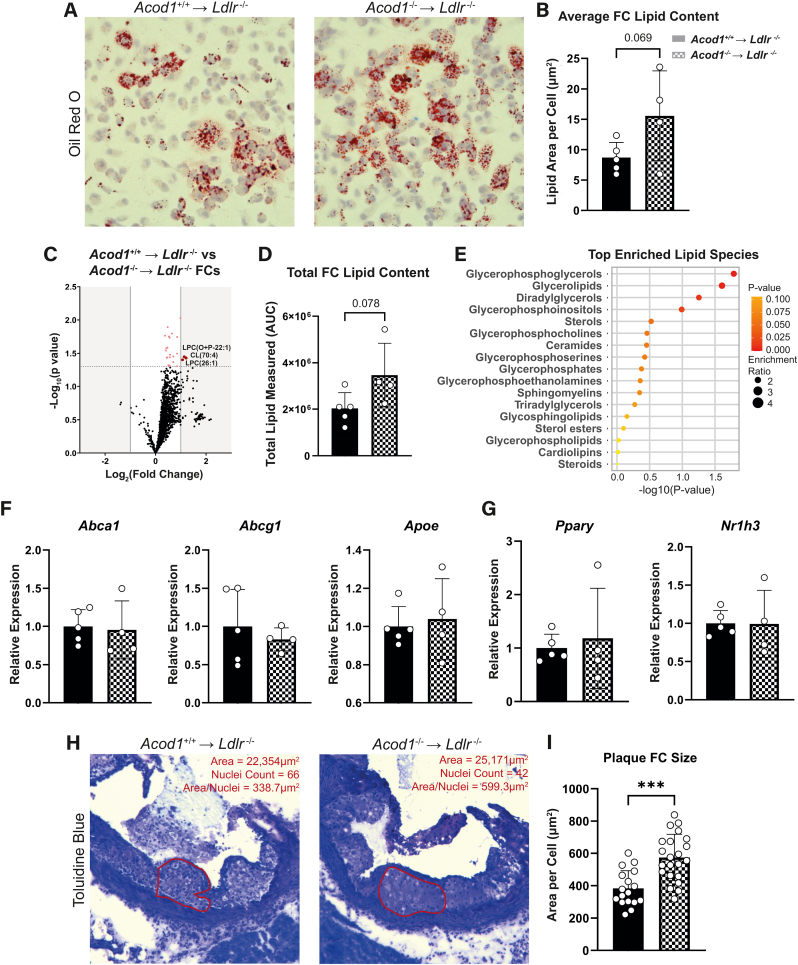
**Itaconate deficiency leads to increased lipid content in foam cells. A)** Oil red O staining of peritoneal foam cells (FCs) from *Acod1*^+/+^ → *Ldlr*^*−/−*^ (left) and *Acod1*^−/−^ → *Ldlr*^*−/−*^ (right) mice, **B)** quantified by calculating lipid area per nucleus stained with hematoxylin (*n* = 5/4 for *Acod1*^+/+^ → *Ldlr*^*−/−*^/*Acod1*^−/−^ → *Ldlr*^*−/−*^, unpaired *t*-test). **C)** Volcano plot of lipidomics from FCs (red = increased in *Acod1*^−/−^ → *Ldlr*^*−/−*^; blue = decreased in *Acod1*^−/−^ → *Ldlr*^*−/−*^; grey = not significant). Dotted line represents significance cut-off (p < 0.05). **D)** Total lipid content measured in all lipid species from lipidomics (*n* = 5/4, unpaired *t*-test). **E)** Lipid species enrichment analysis from the lipidomics dataset. **F)** qPCR-derived gene expression of proteins involved in lipid transport and **G)** their accompanying transcription factors from FCs (*n* = 5/4, unpaired *t*-test). **H)** One of the three aortic root valve cusps in *Acod1*^+/+^ → *Ldlr*^*−/−*^ (left) and *Acod1*^−/−^ → *Ldlr*^*−/−*^ (right) transplanted mice stained with toluidine blue where an area of foam cells (red) was measured and nuclei were counted to **I)** calculate cell size (area/nuclei count; *n* = 17/22, unpaired *t*-test). Data points display individual mice and error bars show SD. *FC* = Foam Cell; *AUC* = Area Under the Curve; *NES* = Normalized Enrichment Score. (For interpretation of the references to colour in this figure legend, the reader is referred to the Web version of this article.)

As FCs are a specific subset of macrophages in atherosclerosis, we tested how *Acod1* deficiency affects these cells. Whilst there is a debate on the inflammatory potential of FCs, their ability to handle excessive lipids is known to be dysregulated which leads to cell death and necrotic core development [31]. Neutral lipid staining by Oil red O revealed a trend of increased lipid accumulation in peritoneal FCs from *Acod1*^−/−^ → *Ldlr*^*−/−*^ atherosclerotic mice compared to wild type littermates (Fig. 3A & B). This increased FC lipid accumulation was confirmed by targeted mass spectrometry lipidomic assay (Fig. 3C & D), where glycerol-containing lipids were the most elevated (Fig. 3E). In order to identify the mechanism behind *Acod1*s’ role in FC lipid homeostasis, we next examined gene expression of typical lipid transport markers. Since target genes of both PPAR-γ and LXR (*Nr1h3*) transcription factors encode proteins that are responsible for cellular lipid transport [[32], [33], [34], [35], [36]], we measured these in the FCs. Nevertheless, expression of lipid transport genes and their transcription factors was similar in the two groups (Fig. 3F & G). Next, we quantified the size of macrophages within the plaque, as excessive lipid content can result in cellular hypertrophy. We found a significant increase in cell size in plaques from the *Acod1*^−/−^ → *Ldlr*^*−/−*^ group (Fig. 3H & I). We thus show that *Acod1*^−/−^ macrophages have a higher lipid content.

### 2.4 Plaque stability is improved by reduced apoptosis and necrotic core development in Acod1^−/−^ mice

**Fig. 4:**
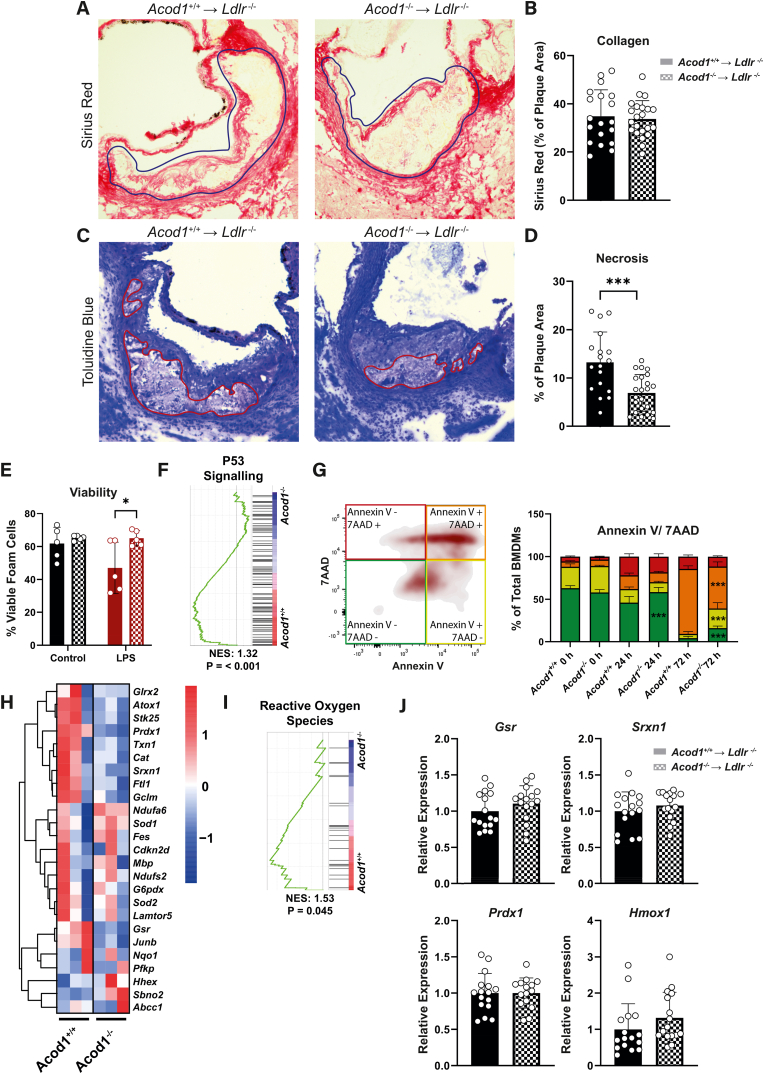
***Acod1* deficiency reduces apoptosis and necrosis. A)** Aortic root segments were stained for Sirius Red to **B)** quantify red collagen staining area as a percentage of the plaque area (blue outline), expressed as an average of the three valve cusps in *Acod1*^+/+^ → *Ldlr*^*−/−*^ (left) and *Acod1*^−/−^ → *Ldlr*^*−/−*^ (right) mice (*n* = 19/22 for *Acod1*^+/+^ → *Ldlr*^*−/−*^/*Acod1*^−/−^ → *Ldlr*^*−/−*^, unpaired *t*-test). **C)** Necrotic core outlined (red) on one of the three aortic root valve cusps stained with toluidine blue which was then **D)** quantified as a percentage of the total plaque and expressed as an average of the three cusps (*n* = 18/22, unpaired *t*-test). **E)** Cell viability in unstimulated (black) or LPS (100 ng/mL) stimulated (red) FCs after 24 h, measured via flow cytometry analysis of fixable viability dye (FVD) eFluor™ 780 (*n* = 5, two-way ANOVA). **F)** P53 signaling pathway enrichment from a gene set enrichment analysis of dataset GSE145950 by Swain et al., 2020 comparing LPS-stimulated *Acod1*^+/+^ and *Acod1*^−/−^ BMDMs. **G)** Flow cytometry quantification of an Annexin V/7AAD apoptosis and necrosis assay on *in vitro* cultured *Acod1*^+/+^ and *Acod1*^−/−^ BMDMs stimulated with LPS (100 ng/mL) over 72 h (*n* = 3, two-way ANOVA). **H)** Gene expression heatmap of reactive oxygen species-related markers and **I)** subsequent gene set enrichment analysis comparing LPS stimulated *Acod1*^+/+^ and *Acod1*^−/−^ BMDMs from dataset GSE145950 by Swain et al., 2020. **J)** qPCR derived gene expression of redox markers from mouse aortic arches (*n* = 15/16, unpaired *t*-test). Gating strategies for flow cytometry is provided in Supplemental Fig. 4. Data points display individual mice and error bars show SD. (For interpretation of the references to colour in this figure legend, the reader is referred to the Web version of this article.)

Considering that both FC formation and monocyte recruitment are two driving factors of plaque instability, we examined markers for plaque vulnerability. Plaques stained with Sirius Red showed no differences in fibrous cap collagen formation between the two groups (Fig. 4A & B). Conversely, when we measured plaque necrotic burden using toluidine blue, we observed that plaques from *Acod1*^*−/−*^ → *Ldlr*^*−/−*^ mice had 50 % decreased necrotic plaque area (Fig. 4C & D). A decrease in plaque necrosis raised the question as to whether *Acod1*^*−/−*^ macrophages had an improved cell survival and were subjected to lower oxidative stress. Therefore, we explored the effect of *Acod1* on cell death in inflammatory conditions, where we detected an increased viability in *Acod1*^−/−^ → *Ldlr*^*−/−*^ FCs (Fig. 4E). Additionally, we performed gene set enrichment analysis (GSEA) on a dataset from Swain et al., 2020, which displayed that *Acod1*^−/−^ BMDMs downregulate the P53 apoptosis pathway (Fig. 4F). Hence, we performed an apoptosis/necrosis assay with 7AAD and Annexin V on BMDMs in inflammatory conditions. We observed that *Acod1*^−/−^ BMDMs were protected from entering late apoptosis after 72 h of activation, resulting in higher percentages of viable cells (Fig. 4G). Since generation of ROS and anti-oxidant responses are known to be regulated by Acod1 and itaconate, we analyzed the expression of ROS pathway genes in the dataset from Swain et al., 2020 (Fig. 4H & I) [[22], [23], [24]]. This analysis indicated that inflammatory *Acod1*^+/+^ BMDMs had higher expression of anti-oxidant genes than *Acod1*^−/−^ BMDMs. Thus, we measured the expression of the most upregulated anti-oxidant genes from this dataset, as well as the key anti-oxidant marker *Hmox1,* in mouse aortic arches (Fig. 4J). Expression of these suppressors of oxidative stress were however similar in both groups. We similarly measured expression of *Ifnb1* as this is described to be regulated by unmodified itaconate however this was unaffected (data not shown). Consequently, *Acod1* deficiency leads to increased plaque stability, which is likely facilitated by reduced macrophage and FC apoptosis, not occurring through differential regulation of anti-oxidant genes *in vivo*.

### 2.5 Shifted glycolytic flux towards glycerol synthesis in Acod1^−/−^ macrophages enables increased *de novo* synthesis of triglycerides

**Fig. 5:**
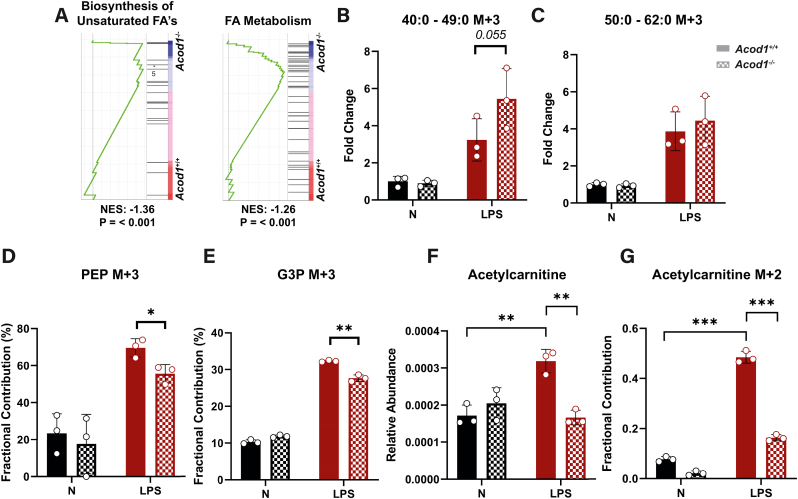
**Broken glycolysis towards triglyceride synthesis may explain increased macrophage lipid content. A)** Gene set enrichment analysis of *de novo* fatty acid (FA) synthesis-related pathways of dataset GSE145950 by Swain et al., 2020. **B)** Total M+3 fraction fold change of triglycerides with carbon content of either 40–49 carbons or **C)** 50–62 carbons from a lipid based fluxomics using ^13^C-glucose isotopic labeling in *Acod1*^+/+^ and *Acod1*^−/−^ BMDMs stimulated with or without LPS (100 ng/ml) for 24 h (*n* = 3, two-way ANOVA). **D)** Fractional contribution of M+3 phosphoenolpyruvate (PEP) and **E)** M+3 glycerol-3-phosphate (G3P) in fluxomics of *Acod1*^+/+^ and *Acod1*^−/−^ BMDMs stimulated with or without LPS (100 ng/ml) for 24 h and cultured with ^13^C-glucose for isotope labelling (*n* = 3, two-way ANOVA). **F)** Relative abundance of metabolite acetylcarnitine from the metabolomics dataset and **G)** the fractional contribution of M+2 acetyl carnitine in the total pool of acetylcarnitine isotopes from the fluxomics dataset (*n* = 3, two-way ANOVA). Data points display individual mice and error bars show SD.

**Supplemental Fig. 5:**
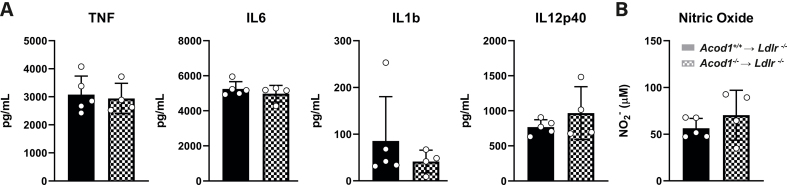
**Heatmap of triglyceride species in inflammatory *Acod1***^**+/+**^**and *Acod1***^**−/−**^**BMDMs.**^13^C-glucose cultured *Acod1*^+/+^ and *Acod1*^−/−^ BMDMs stimulated with (red) or without (black) LPS (100 ng/ml) for 24 h were measured in a fluxomics experiment for labeling of lipid species. Shown are Z-score averages of 3 samples from M+3 fraction fold changes of the displayed lipids (two-way ANOVA).

Since the inflammatory state of FCs was similar between the two groups (Supp. Fig. 5A and 5B), we explored how *Acod1* deficiency affected the metabolic profile of macrophages to explain viability differences. Activated *Acod1*^+/+^ and *Acod1*^*−/−*^ BMDMs were metabolically distinct and displayed enriched purine metabolism, causing metabolites inosine, inosinic acid and hypoxanthine to accumulate in *Acod1*^−/−^ macrophages (Supp. Figs. 6A–D, Supp. Table 1). Next, given that biosynthesis of unsaturated fatty acids (FA) and FA metabolism pathways were enriched in *Acod1*^−/−^ BMDMs, we tested whether the increased lipid content could be coming from *de novo* fatty acid synthesis (Fig. 5A). Thus, we performed a stable isotope tracer-based experiment in BMDMs to study differences in glycolytic contribution towards lipid metabolism using U^13^C-glucose. Fractional contribution of different triglyceride (TG) isotope species showed a trend of TG's with shorter long chain fatty acids (LCFA's) comprising of 40–49 total carbons (Fig. 5B) contained more M+3 labelling in *Acod1*^−/−^ BMDMs which was not observed for TG's with longer LCFA's containing 50–62 total carbons (Fig. 5C & Supp. Fig. 7). This suggested *de novo* lipogenesis of TG species, particularly of TG's containing shorter LCFA's, supporting the previous observation that *Acod1*^−/−^ → *Ldlr*^*−/−*^ FCs contained more total glycerol-containing lipids (Fig. 3E). When looking at glycolytic flux, phosphoenolpyruvate (PEP), the immediate step in glycolysis after synthesis of glycerate derivatives, had lower M+3 fractional contribution (Fig. 5D). Additionally, we detected lower M+3 fractional contribution of glycerol-3-phosphate (G3P) (Fig. 5E). Lower PEP labelling indicates a shift in glycolytic flux, whilst decreased G3P labelling points towards this shift being directed towards glycerol derivative synthesis. Due to the increased M+3 labelling of TG's, we suspect that the glycolytic flux in *Acod1*^*−/−*^ BMDMs is redirected towards TG synthesis. Additionally, we observed that LPS induced an accumulation of acetylcarnitine in *Acod1*^*+/+*^ BMDMs, which remained at a basal level in *Acod1*^−/−^ BMDMs (Fig. 5F). This accumulation was mediated by glucose-derived acetyl-CoA synthesis exhibited by a significant increase in M+2 labelling of acetylcarnitine in activated *Acod1*^+/+^ BMDMs (Fig. 5G). This significant accumulation of acetylcarnitine in *Acod1*^+/+^ macrophages reduces the availability of carnitine to transport lipids for beta-oxidation. Together these data suggest that the observed increased lipid content in *Acod1*^−/−^ macrophages can be partially explained by a shift in glycolytic flux towards synthesis of glycerol derivatives and subsequent *de novo* lipogenesis of TG's.

## 3 Discussion

Since its discovery as an immunomodulatory metabolite, itaconate gained increasing interest as a potential target to manage disease related to chronic inflammation and oxidative stress [37,38]. In this study, we focus on itaconates’ role in the development of atherosclerotic plaques, demonstrating that myeloid-*Acod1* deficiency elevates overall monocyte recruitment and viability, which leads to increased plaque stability by slowing necrotic core growth. Moreover, *Acod1*-deficiency increases lipid-handling capabilities, which may lower the occurrence of aberrantly activated macrophages due to a restored balance of lipid efflux, uptake, biosynthesis and lysis, thus promoting less vulnerable plaques [39]. Consequently, we suggest that ACOD1 should be explored as a potential target in human inflammatory diseases.

Our work exposes the modulating properties of *Acod1* on the migration of circulating monocytes. As such, the patrolling Ly6C^low^ monocyte subset is more abundant in the *Acod1* deficient group and in turn, we observe increased recruitment of monocytes to the lesion site in mice with more stable plaques. This reported increase in circulating Ly6c^low^ monocytes occurring at week 8 post HFD suggests that monocytosis is elicited in response to the high cholesterol diet. No differences in BM composition after sacrifice further supports this and implies that there were no variations in BM composition during transplantation. The role of Ly6C^low^ monocytes and their differentiation into macrophages has been widely shown to be of an anti-inflammatory nature and inversely correlate with plaque growth, whilst Ly6C^high^ monocytes and macrophages are considered pro-inflammatory and promote plaque development [11,[40], [41], [42]]. In another study, Nur77-deficient mice with reduced Ly6c low monocyte numbers promoted the differentiation of Ly6c^high^ monocytes into pro-inflammatory macrophages and resulted in exacerbated atherosclerosis progression [40,41]. These two observations suggest an atheroprotective role for Ly6C^low^-monocyte-derived macrophages within the plaque. Comparatively, the Ly6C^low^ monocytes within the current study are at a higher quantity and monocytes have an improved recruitment capacity, potentially explained by upregulation of *Icam1*, in mice with reduced necrotic core development. Therefore, based on this data and previous research performed on the role of Ly6C^low^ monocytes in plaques, increased circulating Ly6C^*low*^ monocytes when targeting *Acod1* in atherosclerosis is not detrimental to disease progression.

Based on our own and others’ *in vitro* data showing increased inflammatory responses in the absence of Acod1 in macrophages, one might expect a worse atherosclerosis outcome in Acod1-deficient mice [17,38]. A recent paper indeed shows that atherosclerosis induction by PCSK9-AAV administration results in increased inflammation and lesion area in mice that lack ACOD1, and the authors propose an important role of NRF2 in mediating this phenotype. Interestingly, the new *in vivo* data we present here rather shows that Acod1 deficiency is beneficial in atherosclerosis, as highlighted by the decreased necrotic core size. Since this observation was unexpected, we looked further into the phenotype of these macrophages in order to explain how macrophages with an increased inflammatory state could result in more stable plaques. As such, we found that *Acod1* deficient macrophages underwent apoptosis at a lower rate. Considering that apoptosis can contribute to necrotic core development, we believe that cell survival in *Acod1*^*−/−*^ → *Ldlr*^*−/−*^ mice is a viable explanation for the observed reduction in necrotic core [43,44]. Together, the publication by Song et al. and our own research uncover similarities (i.e. increased circulating monocyte levels in the absence of ACOD1), but also important differences (inflammatory status and phenotype of atherosclerotic plaques), and complementary mechanisms (apoptosis and necrosis aspect in our study, and the importance of NRF2 in the work of Song et al.) that should all be taken into account when considering exploiting the IRG1-itaconate axis for atherosclerosis therapy. Differences between both studies might be explained by differences in experimental settings including for example the use of different mouse sources, and the application of bone marrow transplantation towards *Ldlr*^*−/−*^mice versus PCSK9-AAV injection to elicit atherosclerosis.

Given the important role of apoptosis and necrosis, we next wanted to understand the underlying mechanisms that provided *Acod1*^*−/−*^ macrophages with a more resilient survival. Reactive oxygen species, which have been demonstrated to drive apoptosis and plaque progression, are shown in numerous studies to be controlled to some extent by Acod1, where *Acod1* deficiency reduces ROS production [[21], [22], [23]]. Anti-oxidant genes, which are typically upregulated in environments with high ROS content, were expressed lower in *Acod1*^−/−^ macrophages [24]. This indicates that ROS generation in *Acod1*^−/−^ → *Ldlr*^*−/−*^ plaques may be low, however when measuring aortic arch gene expression of top anti-oxidant genes, we were unable to detect a difference between the two groups, regardless of the difference in *Acod1* expression. This could be due to the fact that foam cells within the *Acod1*^−/−^ → *Ldlr*^*−/−*^ plaques contain more lipids and thus more oxLDL, which could upregulate anti-oxidant gene expression to the degree that is similar to *Acod1*^+/+^ → *Ldlr*^*−/−*^ plaques. Additionally, we observed increased expression of anti-inflammatory marker *Il10* within the plaque which is known to lessen plaque development [45,46]. Indeed, the increased *Il10* in our setting could contribute to a more stable plaque however in conjunction with increased *Tnf* it is unclear how effective IL-10 cytokine signalling would be.

In our metabolomics studies, we showed that *Acod1*^+/+^ and *Acod1*^*−/−*^ BMDMs had distinct metabolic profiles, owed in part to metabolites inosine, inosinic acid and hypoxanthine. Previously, a link has been shown between arrested *de novo* synthesis of guanine-containing metabolites and cell apoptosis, since replenishment of guanine pools to fulfil transcription requirements are unmet [47,48]. The larger availability of nucleotide generating metabolites in *Acod1*^*−/−*^ BMDMs may sustain their high demand during inflammatory activation in plaques, possibly explaining the reduced apoptosis in *Acod1*^−/−^ myeloid cells. *Acod1*^+/+^ and *Acod1*^*−/−*^ BMDMs were also in part metabolically distinct given that lipid content of *Acod1*-deficient myeloid cells and plaque cells were enriched. Since myeloid cells display lower apoptosis, this could explain the observation of increased lipid loading in our *in vivo* study, as these cells may be partially protected from excess lipid-induced apoptosis [49,50]. On the lipid metabolism front, we observed an upregulation of glucose utilisation for glycerol synthesis, which can be used in TG synthesis, which was also increased in *Acod1*^−/−^ macrophages. Interestingly, TG synthesis in FCs was shown to enhance their secretion of inflammatory mediators such as prostaglandin E2 (PGE2), which also has been shown to improve macrophage recruitment [51]. It is also an essential part of the cell membrane and thus increased production in *Acod1*^−/−^ macrophages may allow the cell to better handle the cellular hypotrophy induced by lipid uptake in the plaque.

Carnitine, an enzyme responsible for shuttling LCFA's across the mitochondrial membrane for beta-oxidation, was greatly used as an acetyl-CoA sink in inflammatory *Acod1*^*+/+*^ macrophages, generating more acetylcarnitine [52,53]. This phenomenon occurs when glycolytic activity is greater than the TCA cycle, leading to an accumulation of acetyl-CoA. Carnitine in this situation reduces the acetyl-CoA pool [53]. The consequence however is inhibition of carnitine-mediated LCFA transport and subsequent beta oxidation. We observed this through high M+2 acetylcarnitine labelling in *Acod1*^+/+^ macrophages. Moreover, itaconate is another source of acetyl-CoA as it is converted into itaconyl-CoA and subsequently metabolised into acetyl-CoA, only further adding to the pool in inflammatory macrophages [54]. In contrast, itaconate-deficient macrophages have an amplified OXPHOS and thus a larger demand for acetyl-CoA pools, likely explaining the vastly reduced M+2 labelling of acetylcarnitine in *Acod1*^−/−^ macrophages [17]. Therefore, in this paper we hypothesise a new mechanism for the poor lipid handling capability of plaque inflammatory macrophages, mediated by acetyl-CoA blocking carnitine transport of lipids and subsequent beta-oxidation (Fig. 6).

**Fig. 6:**
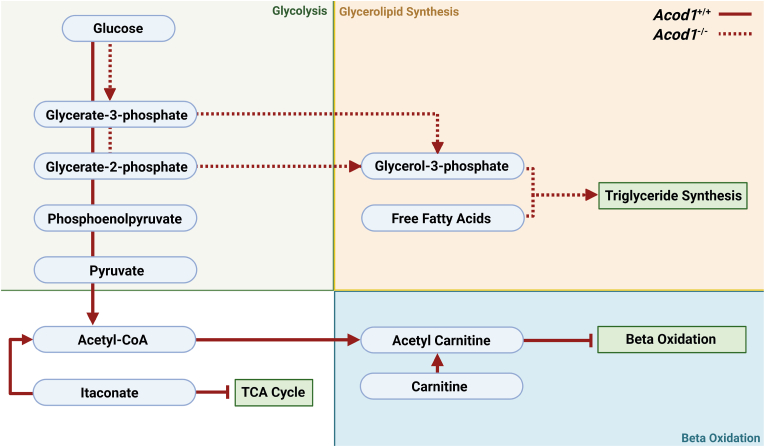
**Glycolytic flux in Acod1**^**+/+**^**and Acod1**^**−/−**^**inflammatory BMDMs.** Schematic of glucose flux in inflammatory (LPS) *Acod1*^+/+^ (solid red) and *Acod1*^−/−^ (dashed red) BMDMs. Arrows display where differences in glucose flux occur between the two groups but are not the only flux occurring. Blue curved boxes are metabolites and green straight boxes are pathways. Created with BioRender.com.(For interpretation of the references to colour in this figure legend, the reader is referred to the Web version of this article.)

Based on the data provided in our study, we propose that silencing the Acod1-itaconate axis in atherosclerosis leads to a favourable plaque phenotype. Total circulating Ly6C^Low^ monocytes and monocyte recruitment to the plaque are increased, whilst plaque FC viability is also increased, owed in part to re-wired glycolytic flux towards TG synthesis. As such, bone marrow Acod1-deficiency reduces necrotic core development in plaques. Yet, it should be noted that another recent publication that employs an alternate way of atherosclerosis induction rather shows that Acod1 knockout elicits increased inflammation and larger plaques. Clearly our studies warrant future research into the potential use of the ACOD1-itaconate axis to manage atherosclerosis progression. The generation of ACOD1-targeting small molecules (both inhibitory and activating ones) will help to further uncover the potential benefit of modulating ACOD1/itaconate to improve disease outcome.

## 4 Materials & methods

### 4.1 Mice

Bone marrow obtained from Wild-type C57BL/6J(c) 8- to 16-week-old mice originated from Charles River Laboratories and were housed in the Universitair Proefdiercentrum, Vrije Universiteit Amsterdam and terminated by CO_2_ asphyxiation. Bone marrow from female *Acod1*^+/+^ and *Acod1*^−/−^ mice on a C57BL/6 N background, aged between 8 and 13 weeks, housed at the SPF animal facility at the University of Luxembourg and terminated by cervical dislocation, were generously gifted from Dr. Alessandro Michelucci (Luxembourg Institute of Health). Using embryonic stem cells (ESC) from the Knockout Mouse Project Repository (KOMP, University of California, DAVIS), Dr. Haruhiko Koseki produced the Irg1^tm1a(KOMP)Wtsi^ strain at the RIKEN Institute [55]. Briefly, recipient female C57BL/6 N blastocysts were injected with *Acod1*^−/−^ C57BL/6 N ESCs and subsequently bred with wild-type C57BL/6 N mice. *Acod1*^−/−^ mice and *Acod1*^−/−^ C57BL/6 N littermate controls were then generated by crossing heterozygote mice. *Acod1*^+/+^ and *Acod1*^−/−^ were then phenotyped using qPCR. For atherosclerosis studies, low density lipoprotein receptor (*Ldlr*) KO mice with a C57BL/6 background (Jackson laboratories) were used. All mice were housed in 12 h light/dark cycle and had free access to sterile food and water. All experiments were approved by the Committee for Animal Welfare under license number AVD118002016750 (Amsterdam UMC, Amsterdam, The Netherlands).

### 4.2 Human donors

Human monocytes were harvested from anonymous donor buffy coats provided by Sanquin blood bank in Amsterdam, The Netherlands. All donors had granted permission to use material for medical research through written informed consent and all experiments using such material adhered to the Declaration of Helsinki.

### 4.3 Bone marrow isolation, differentiation and stimulation

Bone marrow of C57BL/6 or *Acod1*^+/+^ and *Acod1*^−/−^ mice was isolated by flushing femurs and tibias. BMDMs were then generated by culturing bone marrow for 6 days in RPMI-1640 medium supplemented with 10 % FCS, 2 mM l-glutamine (Thermo Fisher Scientific), 100 U/mL penicillin-streptomycin (Thermo Fisher Scientific) and 15 % L929 conditioned medium. BMDMs were then plated at a density of 1 x 10^6^/mL and pre-treated with 5 mM unmodified itaconate (Sigma-Aldrich) for 3 h in itaconate related functional experiments. To elicit an inflammatory response and to activate macrophages, BMDMs were subsequently treated with 100 ng/mL of lipopolysaccharide (LPS from *Escherichia coli*; O111:B4; Sigma) as indicated (3, 24 or 72 h). For fluxomics experiments, isotopic labelling was achieved by supplementing glucose-free medium with U^13^C-glucose, as well as previously mentioned supplementations, for 12 h prior to stimulations.

### 4.4 Human monocyte isolation, differentiation and activation

Human monocytes were harvested using Ficoll based density gradient separation of PBMC's followed by isolation of CD14^+^ monocytes with the use of magnetic beads (Miltenyi). Monocytes were then seeded in plates at a density of 1 x 10^6^/mL and cultured for 6 days in Iscove's Modified Dulbecco's Medium (IMDM) medium supplemented with 10 % FCS, 2 mM l-glutamine (Thermo Fisher Scientific), 100 U/mL penicillin-streptomycin (Thermo Fisher Scientific) and 50 ng/mL human M-CSF (Miltenyi). On day 3 and 6 of culturing, old medium was replaced with fresh medium with the same supplements. Differentiated macrophages were stimulated with either 5 mM itaconate or 10 ng/mL LPS as described above.

### 4.5 Atherosclerosis *in vivo*

Atherogenic *Ldlr*^−/−^ female mice were irradiated with a lethal amount of 6 Gy on 2 consecutive days. Within each cage containing 5 mice, mice were randomly selected to be intravenously transplanted with 5 x 10^6^ cells of either *Acod1*^+/+^ or *Acod1*^−/−^ bone marrow, with no cage containing less than 2 mice transplanted with the same bone marrow. This resulted in 22 *Acod1*^+/+^ → *Ldlr*^*−/−*^ and 23 *Acod1*^−/−^ → *Ldlr*^*−/−*^ mice. After 6 weeks of bone marrow reconstitution, regular chow diet was substituted for HFD (15 % fat and 0.15 % cholesterol, Ssniff Spezialdiäten) for the remaining 9 weeks of the experiment. Roughly 100uL of blood was isolated via tail incision on weeks 0, 5 and 8. 4 days prior to sacrifice, a subset of 5 vs 5 *Acod1*^+/+^ → *Ldlr*^*−/−*^ and *Acod1*^−/−^ → *Ldlr*^*−/−*^ mice were intraperitoneally injected with 3 % thioglycollate, to induce a sterile inflammation. On week 9, mice were terminated by CO_2_ asphyxiation. Hearts were punctured immediately after sacrifice to drain mice of blood for storage of plasma and quantifying engraftment through measurement of *Ldlr* expression by qPCR. Mice injected with thioglycollate received a peritoneal lavage with ice cold PBS and isolated FCs were placed in RPMI-1640 culture medium supplemented with 10 % FCS, 2 mM l-glutamine, 100 U/mL penicillin-streptomycin. Hearts were then removed and stored in tissue-tek (DAKO) for histological staining whilst spleens were halved, to be immediately used for leukocyte quantification as well as storage in tissue-tek. Bone marrow was isolated from a subgroup of 5 vs 5 *Acod1*^+/+^ → *Ldlr*^*−/−*^ and *Acod1*^−/−^ → *Ldlr*^*−/−*^ mice for precursor quantification.

### 4.6 Blood and spleen parameters

Spleens were weighed and crushed through a 45 μm filter and then stained for leukocyte markers as stated in Supplemental Table 2 and measured by flow cytometry. Leukocyte composition was normalized on spleen weight. Blood plasma was used to enzymatically determine cholesterol and triglyceride levels according to the manufacturer's protocol (Roche). Leukocyte quantification was performed on blood after 3 steps of erylysis treatment. 10 μL of blood was used to quantify CD45^+^ cell content per mL. Leukocyte subsets were assessed by flow cytometry using antibody stains as stated in Supplemental Table 2 and percentages were used to calculate subset per mL of blood from quantified CD45^+^ cells per mL.

### 4.7 Histology

Aortic root segments were cut from tissue-tek stored organs using the Leica CM3050S cryostat at −20 °C. Aortic roots sections were then either stained for Toluidine Blue (0.2 % in PBS, Sigma-Aldrich) to quantify plaque size and necrotic burden or with Sirius Red (30 min, 0.05 % Direct Red in saturated picric acid, Sigma) to measure plaque collagen levels using Adobe Photoshop software. Aortic root segments were also fixated using dry acetone and blocked with Avidin/Biotin Blocking Kit (Vector Laboratories) before staining for ER-MP58 and secondarily stained with biotin-labeled rabbit anti-rat antibody. ER-MP58 binding was amplified using ABC kit (Vector Laboratories) in combination with ImmPACT AMEC Red Peroxidase Substrate kit (Vector Laboratories). Plaque parameters were determined by our expert pathologist. Plaque size was calculated as a total area of all 3 aortic root valves from the average of 4 consecutive slides, whilst necrosis and collagen content was expressed as a percentage of this total area. Plaque cell size was calculated in Adobe Photoshop software by counting nuclei within a defined and measured area. ER-MP58 staining was quantified by counting luminal positively stained cells.

### 4.8 Flow cytometry

To quantify bone marrow precursor populations and BMDM/FC viability, cells were blocked with anti-CD16/CD32 Fc-block in a V-bottom plate. Subsequent stains against cell surface markers for identifying bone marrow precursors or FC viability are displayed in Supplemental Table 2. BMDM viability was measured by first staining for Annexin V-FITC diluted in Annexin V binding buffer before staining with 7AAD. Stained cells were measured on a Beckman Coulter CytoFLEX or BD LSR II Fortessa and analyzed using FlowJo (Tree Star).

### 4.9 Gene expression analysis

RNA was isolated from either BMDMs or FCs cultured at a density of 0.5 x 10^6^ per well in 24 well plates using the GeneJET RNA Purification Kit (Thermo Fisher Scientific) or aortic arches using RNeasy Mini Kit (Qiagen). Aortic arches were homogenized in Trizol and RNA was extracted using chloroform to layer the RNA content. RNA from plaques were subsequently added to RNeasy columns and DNase I was added on top prior to further wash and spin steps. An Implen nanodrop N60 was used to quantify the RNA and then was reverse-transcribed into cDNA using the High-Capacity cDNA Reverse Transcription Kit (ThermoFisher). cDNA was used for gene expression analysis in a qPCR with SYBR Green Fast mix dye (Applied Biosystems) using the ViiA 7 system (Applied Biosystems). *Cycloa* and *Rplp0* were used as housekeeping genes to normalize expression of measured genes. Primer sequences can be found in Supplemental Table 3.

### 4.10 Transcriptomics

Fastq files were extracted using experiment accession GSE145950 from Gene Expression Omnibus (GEO). Reads were trimmed using *Trimmomatic* and then aligned to the mouse genome mm10 using *RNAStar*. Mapped reads in the BAM format were assigned to genomic features with *featureCounts*, providing feature base lengths alongside to calculate reads per kilobase million (RPKM) values. Differential gene expression was subsequently derived by means of the *DESeq2* package. The resulting data was then run through the GSEA program (University of California San Diego) against KEGG gene sets to identify differentially regulated pathways.

### 4.11 Fluxomics

Fluxomics was performed by combining a fluxomics method with a metabolomics and lipidomics method [56,57]. To a 2 mL tube, containing BMDMs, solvents were added to achieve a total volume of 2 mL containing 1 mL of chloroform, 500 μL of methanol and 425 μL of water. All samples were mixed thoroughly, before centrifugation for 10 min at 14.000 rpm.

For polar fluxomics, the visible polar phase on the top layer was pipetted into a 1.5 mL tube and dried at 60 °C with a vacuum concentrator. 100 μL 6:4 (v/v) methanol:water was added to dried samples for reconstitution. A Waters Acquity ultra-high performance liquid chromatography system in combination with a Bruker Impact II™ Ultra-High Resolution Qq-Time-Of-Flight mass spectrometer was used to analyze metabolites. Subsequent sample acquisition was performed as previously described [58]. Bruker TASQ software version 2.1.22.3 was used to analyze the data. Accurate mass, ion mobility data, (relative) retention times and fragmentation spectra was used to identify metabolites. For fluxomics, relative isotope contribution was calculated using IsoCorrectoR Release 3.13 [[59], [60]].

For lipid fluxomics, the visible phase on the bottom layer of the liquid-liquid extraction was pipetted in to a clean 1.5 mL tube and dried at 60 °C with a nitrogen concentrator. 100 μL of 1:1 (v/v) methanol:chloroform was added to the dried sample to dissolve it. A Thermo Scientific Ultimate 3000 binary HPLC in combination with a Q Exactive Plus Orbitrap mass spectrometer was used to analyze lipids in the samples. Subsequent sample acquisition was performed as previously described [61]. Matlab was used to write an in-house pipeline and analyze data (https://www.mathworks.com) by annotating the monoisotopic lipid peaks and corresponding ^13^C isotopes [62]. Accurate mass, (relative) retention times, analysis of samples with known metabolic defects, fragmentation spectra and relevant standards was used to identify lipids.

### 4.12 Metabolomics

Metabolomics was performed as previously described, with minor adjustments [58,63]. 75 μL of internal standard was combined with 1 mL of chloroform, 500 μL of methanol and 425 μL of water to add to each sample containing cells, which was then mixed and centrifuged for 10 min at 14.000 rpm. The visible polar phase on the top layer was pipetted into a 1.5 mL tube and dried at 60 °C with a vacuum concentrator. The subsequent sample preparation and mass spectrometry method are as described for polar fluxomics. Internal standards with comparable retention times and response in the MS was used to normalize all metabolite intensities, and subsequently to total metabolite content. Accurate mass, ion mobility data, (relative) retention times and fragmentation spectra was used to identify metabolites.

### 4.13 Lipidomics

Lipidomics sample preparation was performed as previously described [64,65]. Briefly: 1:1 (v/v) methanol:chloroform was used to dissolve internal standards and then added to a 2 mL tube containing BMDMs before centrifugation for 10 min at 14.000 rpm. Supernatant was pipetted in to a clean 1.5 mL tube and dried at 60 °C with a nitrogen concentrator. 100 μL of 1:1 (v/v) methanol:chloroform was added to the dried sample to dissolve it. A Thermo Scientific Ultimate 3000 binary HPLC in combination with a Q Exactive Plus Orbitrap mass spectrometer was used to analyze lipids in the samples. Subsequent sample acquisition was performed as previously described [61]. R programming language was used to write an in-house pipeline and analyze data (http://ww.r-project.org). Internal standards for each lipid class were used to normalize reported lipids. Accurate mass, (relative) retention times, analysis of samples with known metabolic defects, fragmentation spectra and relevant standards was used to identify lipids.

### 4.14 Oil red O cell culture

Peritoneal FCs were cultured at a semi-confluent density in Ibidi glass bottom 8-well culture slides and fixed in 4 % formalin. After fixation, cells were incubated with 60 % isopropanol followed by staining with Oil red O (0.3 % in a 3:2 isopropanol:water solution). FCs were then washed with more 60 % isopropanol and subsequently stained with hematoxylin. VectaMount was subsequently used to mount the cells for imaging. Neutral lipid content and nuclei count was analyzed using Image J to quantify lipid area per cell.

### 4.15 Cytokine and NO synthesis

IL6 and TNFα cytokine content in cell culture supernatant was measured using ELISA kits (Invitrogen) following the manufacturers protocol. Secreted NO was measured by NO_2_^−^ quantification using a Griess reagent reaction (Sigma).

### 4.16 Statistics

Data were evaluated with GraphPad Prism 9 using either t-tests or two-way ANOVA. Values are displayed as mean ± SEM of one representative experiment unless otherwise stated. Statistical significance is displayed as ∗ p < 0.05, ∗∗ p < 0.01 and ∗∗∗ p < 0.001 whilst a statistical trend of p < 0.1 and >0.05 is displayed as a number.

## Funding

MdW is supported by Amsterdam UMC, Amsterdam Cardiovascular Sciences, ZonMW (ZonMW Open competition 09120011910025), NWO (Epi-Guide-Edit - KICH1.ST01.20.045) and the European Union (Doctoral Network MIRACLE).

## CRediT authorship contribution statement

**Karl J. Harber:** Writing – review & editing, Writing – original draft, Visualization, Methodology, Investigation, Formal analysis, Conceptualization. **Annette E. Neele:** Writing – review & editing, Methodology, Investigation. **Cindy PAA. van Roomen:** Methodology, Investigation. **Marion JJ. Gijbels:** Writing – review & editing, Methodology, Investigation. **Linda Beckers:** Methodology, Investigation. **Myrthe den Toom:** Methodology, Investigation. **Bauke V. Schomakers:** Writing – review & editing, Methodology, Investigation. **Daan AF. Heister:** Investigation. **Lisa Willemsen:** Writing – review & editing, Investigation. **Guillermo R. Griffith:** Investigation. **Kyra E. de Goede:** Investigation. **Xanthe AMH. van Dierendonck:** Writing – review & editing, Investigation. **Myrthe E. Reiche:** Writing – review & editing, Methodology. **Aurélie Poli:** Investigation. **Frida L-H Mogensen:** Investigation. **Alessandro Michelucci:** Writing – review & editing. **Sanne GS. Verberk:** Writing – review & editing, Investigation. **Helga de Vries:** Writing – review & editing. **Michel van Weeghel:** Writing – review & editing, Funding acquisition, Conceptualization. **Jan Van den Bossche:** Writing – review & editing, Funding acquisition, Conceptualization. **Menno PJ. de Winther:** Writing – review & editing, Funding acquisition, Conceptualization.

## Declaration of competing interest

The authors declare that they have no known competing financial interests or personal relationships that could have appeared to influence the work reported in this papr.

## Figures and Tables

**figs6:**
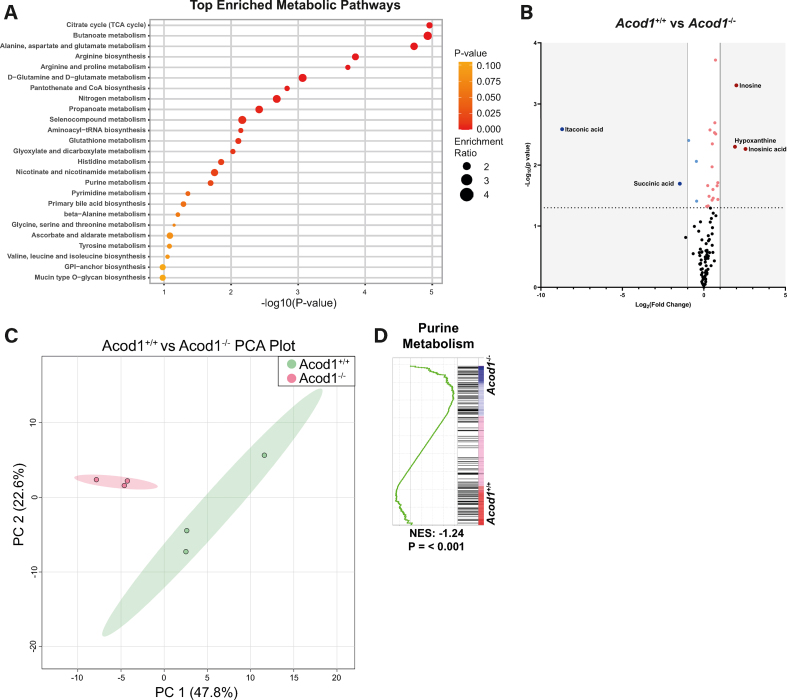


**figs7:**
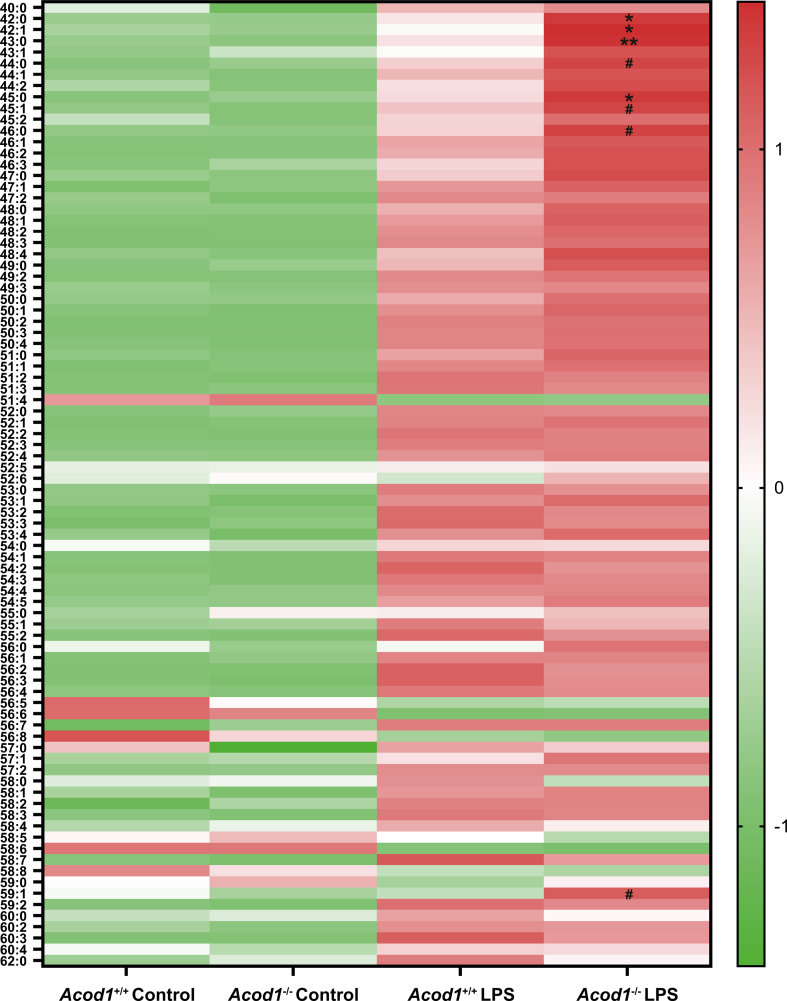


## Data Availability

Data will be made available on request.
